# Cyclosporine A Enhances Th2 Bias at the Maternal-Fetal Interface in Early Human Pregnancy with Aid of the Interaction between Maternal and Fetal Cells

**DOI:** 10.1371/journal.pone.0045275

**Published:** 2012-09-27

**Authors:** Hai-Lan Piao, Song-Cun Wang, Yu Tao, Rui Zhu, Chan Sun, Qiang Fu, Mei-Rong Du, Da-Jin Li

**Affiliations:** 1 Laboratory for Reproductive Immunology, Hospital and Institute of Obstetrics and Gynecology, Fudan University Shanghai Medical College, Shanghai, China; 2 Department of Obstetrics and Gynecology, Hainan Medical College Affiliated Hospital, Haikou, China; University of Medicine and Dentistry of New Jersey, United States of America

## Abstract

Our previous study has demonstrated that cyclosporine A (CsA) administration *in vivo* induces Th2 bias at the maternal-fetal interface, leading to improved murine pregnancy outcomes. Here, we investigated how CsA treatment *in vitro* induced Th2 bias at the human maternal-fetal interface in early pregnancy. The cell co-culture *in vitro* in different combination of component cells at the maternal-fetal interface was established to investigate the regulation of CsA on cytokine production from the interaction of these cells. It was found that interferon (IFN)-γ was produced only by decidual immune cells (DICs), and not by trophoblasts or decidual stromal cells (DSCs); all these cells secreted interleukin (IL)-4, IL-10, and tumor necrosis factor (TNF)-α. Treatment with CsA completely blocked IFN-γ production in DICs and inhibited TNF-α production in all examined cells. CsA increased IL-10 and IL-4 production in trophoblasts co-cultured with DSCs and DICs although CsA treatment did not affect IL-10 or IL-4 production in any of the cells when cultured alone. These results suggest that CsA promotes Th2 bias at the maternal-fetal interface by increasing Th2-type cytokine production in trophoblasts with the aid of DSCs and DICs, while inhibiting Th1-type cytokine production in DICs and TNF-α production in all investigated cells. Our study might be useful in clinical therapeutics for spontaneous pregnancy wastage and other pregnancy complications.

## Introduction

Successful pregnancy requires delicate harmony between fetus-derived trophoblast cells and mother-derived decidual cells. These cells synchronize to establish a unique maternal-fetal immune milieu via the production of regulatory factors, which contribute to fetal survival and development in the maternal uterus until parturition [1], [2], [3]. Cytokines appear to play an important role in modulating and integrating immune and endocrine systems for the maintenance of maternal-fetal tolerance and sufficient placentation. It has been suggested that in a successful pregnancy, a Th2 bias is present both at the maternal-fetal interface and in the periphery; whereas in an abortion-prone pregnancy, a Th1 bias is found both systemically and at the maternal-fetal interface [4], [5], [6]. Furthermore, maternal administration of the immunoregulatory cytokine interleukin (IL)-10 or blockade of tumor necrosis factor (TNF)-α activity was shown to prevent pregnancy loss induced by lipopolysaccharide administration [7], [8]. Thus, the maintenance of the Th2-predominant environment both at the maternal-fetal interface and in the periphery is considered an important target in the treatment of unexplained pregnancy loss.

Cyclosporin A (CsA), a powerful immunosuppressant, that is widely used to prevent organ rejection and to treat several autoimmune diseases such as rheumatoid arthritis, nephrotic syndrome, and systemic lupus erythematosus [9], [10], [11], [12]. CsA suppresses the activation of lymphokine genes essential for T-cell proliferation by disrupting calcium-dependent signal transduction pathways in leukocytes [13]. In addition to this classical mode of action on leukocytes, CsA also exerts different effects on other types of cells, and regulates disparate biological functions [14], [15], [16], [17], [18], [19]. Our previous studies on mice have demonstrated that the administration of CsA (0.1, 1.0, 10 mg/kg) during the window of implantation reduces the embryo resorption rate of CBA/J×DBA/2 abortion-prone matings, by protecting the fetus from maternal immune attack and increasing murine cytotrophoblast cell viability and invasion [19], [20]. These observations suggest that CsA can be used as an effective therapeutic for some pregnancy complications, such as spontaneous abortion. However, the regulatory mechanism underlying the effect of CsA on the Th2 bias at the maternal-fetal interface remains poorly understood.

The main cell types present at the maternal-fetal interface include embryo-derived trophoblasts, decidual stromal cells (DSCs), and lymphocytes. The interplay between these cell types results in the production of various cytokines involved in the Th2 bias in the maintenance of normal pregnancy [6], [21]–[24]. Therefore, we propose that CsA might regulate the interactions among these cells and contribute to the Th2-predominant milieu at the maternal-fetal interface in human early pregnancy. In our present study, we first examined the kinetic secretion of Th1- and Th2-type cytokines in the supernatant of these functional cells at the maternal-fetal interface in human early pregnancy by enzyme-linked immunosorbent assay (ELISA). Thereafter, we investigated the effect of CsA on the interaction and cytokine production in different co-cultures of these cells. Our study demonstrates the complicated crosstalk at the maternal-fetal interface, and the integral regulatory effects of CsA on maternal immunocompetent cells, DSCs and embryonic trophoblasts. These findings might be useful in clinical therapeutics for pregnancy wastage and other pregnancy complications.

## Materials and Methods

### Human Placental and Decidual Tissue Collection

The first-trimester human villi and decidual tissues were obtained from 29 healthy women in early pregnancy confirmed by ultrasound (age, 27.48±4.58 years; gestational age at sampling, 51.93±6.53 days, mean ± SD) whose pregnancies were terminated for non-medical reasons. All the tissues were immediately collected into ice-cold Dulbecco’s modified Eagle medium (DMEM) with high D-glucose or DMEM/F12 (Gibco, Grand Island, NY, USA), transported to the laboratory within 30 min after surgery, and washed in calcium- and magnesium-free Hanks balanced salt solution (HBSS) for trophoblasts or DSC isolation. All procedures involving participants in the study were approved by the Human Research Ethics Committee of Obstetrics and Gynecology Hospital, Fudan University, and all subjects signed a written consent for the collection of tissue samples.

### Isolation and Primary Culture of Human First-trimester Trophoblast Cells

The first-trimester human placentas were separated carefully from the deciduas under a stereomicroscope, pooled and minced into small fragments, then treated by repeated trypsin digestions and Percoll gradient centrifugation according to our previous method [1], [6], [24]. Briefly, the placental tissues obtained from 7–8 separate individuals were pooled and digested by 0.25% trypsin (Bio Basic Inco, BBI, Ontario, Canada) and 0.02% DNase type I (Sigma, Saint Louis, Missouri, USA) at 37°C with gentle agitation for 5 min. The liquid suspension was discarded and the residual tissue was subjected to four cycles of 10-min digestion with the same enzymes. Trypsin digestion in each time was stopped with 10% fetal bovine serum (FBS, Hyclone, Logan, UT, USA) and the liquid digest was harvested. The 4 digests were pooled, centrifuged at 300×*g* for 10 min, and the pellet resuspended in 4 ml DMEM with high D-glucose (Gibco, Grand Island, NY, USA). This suspension was carefully layered over a discontinuous Percoll gradient consisting of 70% to 5% Percoll (vol/vol) in 5% steps of 2 ml each formed by diluting 90% Percoll with HBSS. After centrifugation at 1000×*g* for 20 min, the cells sedimenting at densities between 1.048 and 1.062 g/ml were collected, and washed with DMEM-high glucose medium. These semi-purified cytotrophoblast cells were then diluted and maintained in DMEM-high glucose complete medium (2 mM glutamine, 25 mM HEPES, 100 UI/ml penicillin and 100 mg/ml streptomycin), supplemented with 15% heat-inactivated fetal bovine serum (FBS, Hyclone, Logan, UT, USA) and incubated in 5% CO_2_ at 37°C. The isolated human trophoblast cells were plated in plastic Petri dishes and incubated at 37°C for 30 min to allow the contaminating macrophages to adhere to the plastic. The nonadherent trophoblast cells were transferred to fresh plates as well as eight-chambered LabTek slides at a concentration of 2–4×10^5^ cells/ml, and cultured in DMEM-high glucose complete medium supplemented with 15% heat-inactivated FBS at 37°C in 95% air and 5% CO_2_. This method supplies a 95% purity of trophoblast cells. The characterization and purity of trophoblast cells has been described in details in our previous publications [1], [6], [24].

### Isolation and Primary Culture of DSCs and Decidual Immune Cells (DICs)

DSCs and DICs were isolated by trypsin-DNase I digestion and discontinuous Percoll gradient centrifugation, as described in our previous study [1], [6], [24]. DSCs, which ranged in density between 1.042 and 1.062 g/mL, were recovered and cultured in DMEM/F12 complete medium supplemented with 10% FBS, 100 U/mL penicillin, and 100 µg/mL streptomycin in 5% CO_2_ at 37°C. After primary culture for 30 min at 37°C in 5% CO_2_, non-adherent lymphocytes were removed by washing, leaving DSCs that were 98% pure. Our previous publication describes the characterization of DSCs that were cultured *in vitro* [1]. DICs, which ranged in density between 1.062 and 1.077 g/mL, were collected and cultured in RPMI 1640 complete medium supplemented with 10% FBS, 100 U/mL penicillin, and 100 µg/mL streptomycin in 5% CO_2_ at 37°C. After primary culture for 30 min at 37°C in 5% CO_2_, the adherent DSCs were removed by washing, leaving DICs that were 98% pure.

### Primary Culture of Trophoblasts, DSCs and DICs

The first-trimester trophoblasts, DSCs, and DICs isolated as described above were seeded in 24-well plates (600 µL/well) at a density of 2×10^5^ cells/mL, and the supernatants were collected after 24, 48, and 72 h of culture. Each supernatant was harvested and centrifuged at 2000×*g*, then was removed to a new tube and stored at −80°C.

### Co-culture of Primary Trophoblasts, DSCs and DICs

Freshly isolated trophoblasts, DSCs, and trophoblasts with DSCs (1∶1) were seeded at a density of 1×10^6^ cell/mL per well in 6-well plates overnight. The supernatants were discarded and the wells were washed with 1× phosphate-buffered saline (PBS). The same number of DICs was added into each well, and the cells were cultured continuously for 48 h. DICs were cultured alone as a control, and some wells were then treated with CsA (1.0 µM) or dimethyl sulfoxide (DMSO; vehicle control) (v/v = 1∶1000). The supernatants were collected and centrifuged at 2000×*g*, then removed to a new tube and stored at −80°C for the extracellular cytokine quantitation assay. The cells were harvested for intracellular cytokine analysis by flow cytometry (FCM).

### Immunofluorescence

After 24 h of co-culture, the floating decidual immune cells were harvested for the characterization by FCM. The attached cells including trophoblast cells and DSCs were washed and fixed with phenolformaldehyde (4%; 20 min, 4°C), washed with PBS and permeabilized (10 min, 4°C) with 0.2% Triton X-100 in PBS. Samples were blocked with 2% BSA (in PBS) followed by incubation (overnight, 4°C) with primary Abs. Mouse anti-human cytokeratin (CK) 7 and rabbit anti-human vimentin mAb (Cell Signalling) were used as markers for identification of trophoblast and DSC, respectively. The dilutions of anti-CK 7 and anti-vimentin were both 1/50. Isotype-matched irrelevant IgG (Sino-America) was used as a control. After incubation with primary Ab, the cells were washed in PBS-0.1% Tween 20, and then incubated with fluorescein-isothiocyanate (FITC)-conjugated mouse and phycoerythrin (PE)-conjugated rabbit secondary Abs. After washing, DAPI nuclear stain was added to the cells, which were then washed and mounted with Vectashied (Vector, Burlingame, CA, USA). Florescence images were captured by Leitz DMRX microscope. The experiments were repeated three times.

### Cytokine Quantitation in the Supernatant by ELISA

To detect cytokine secretion, trophoblasts, DSCs, and DICs (1×10^6^/well) were co-cultured with phorbol myristate acetate (PMA, 25 ng/mL) and ionomycin (1 µg/mL) for 4 h in 6-well flat-bottom plates (Nunc, Roskilde, Denmark) before the end of the 48-h culture, with or without CsA. Following this, the levels of cytokines such as IL-4, IL-10, TNF-α, and IFN-γ in the supernatant from each indicated group were quantified using the commercially available Quantikine kit (R&D Systems, Minneapolis, MN, USA) following the manufacturer’s instructions. The lowest limits of detection of the cytokine ELISA kits were as follows: IL-4, 2.0 pg/mL; IL-10, 4.0 pg/mL; IFN-γ, 2.0 pg/mL; and TNF-α, 5.1 pg/mL.

### Monoclonal Antibodies

FITC-conjugated antibodies against IFN-γ (mouse IgG1), CD45 (mouse IgG1), and CK 7 (mouse IgG2b); PE-conjugated antibodies against TNF-α (mouse IgG1), vimentin, and IL-4 (mouse IgG2b); PE-Cy5.5-conjugated antibodies against IL-4 (mouse IgG1); allophycocyanin (APC)-conjugated mAbs against IFN-γ and IL-10 (mouse IgG2b); and corresponding isotype controls were purchased from Caltag Laboratories, Inc. (Burlingame, CA, USA).

### Flow Cytometry

To detect intracellular cytokine production, trophoblasts, DSCs, and DICs (1×10^6^/well) were co-cultured with PMA (25 ng/mL), ionomycin(1 µg/mL), and brefeldin A (10 µg/mL) for 4 h in 6-well flat-bottom plates (Nunc, Penfield, NY, USA) before the end of the 48-h culture with or without CsA. After that, the suspended cells (DICs) and attached cells (trophoblasts and DSCs) were harvested separately, resuspended in PBS at a density of 2×10^6^/mL, and added in 100-µL aliquots to individual Falcon 2054 polystyrene round-bottom tubes (Becton Dickinson, Franklin Lakes, NJ, USA) for immunolabeling. The leukocytes in suspension were washed twice after immediate staining with a mAb against CD45 (a common marker for leukocytes) by a standard immunofluorescence assay, and then were fixed, permeabilized, and stained using TNF-α, IFN-γ, IL-4, and IL-10 mAbs following a 30-minute incubation at 4°C. The attached trophoblasts and DSCs were fixed, permeabilized, and stained for CK7 (a marker for trophoblasts), vimentin (a marker for DSC), TNF-α, IFN-γ, IL-4, and IL-10 mAbs, and then washed twice and resuspended in PBS for FCM analysis. In parallel, isotypic IgG antibodies were used as controls. Samples were analyzed in FACSCalibur flow cytometer (Becton Dickinson) using CellQuest software (Becton Dickinson). Statistical analysis was conducted by using isotype-matched controls as the reference. Typically, less than 1% of positive cells were allowed beyond the statistical marker in the appropriate control.

### Statistical Analysis

Statistical comparisons for the cytokines were evaluated by one-way or two-way analysis of variance. The post hoc Dunnett *t* test was used to compare significance levels between the control and various treatments. All error bars in figures indicate standard error (SE). Statistical significance was accepted at *P*<0.05.

## Results

### Cytokine Secretion of Component Cells at Human Maternal-fetal Interface

**Figure 1:**
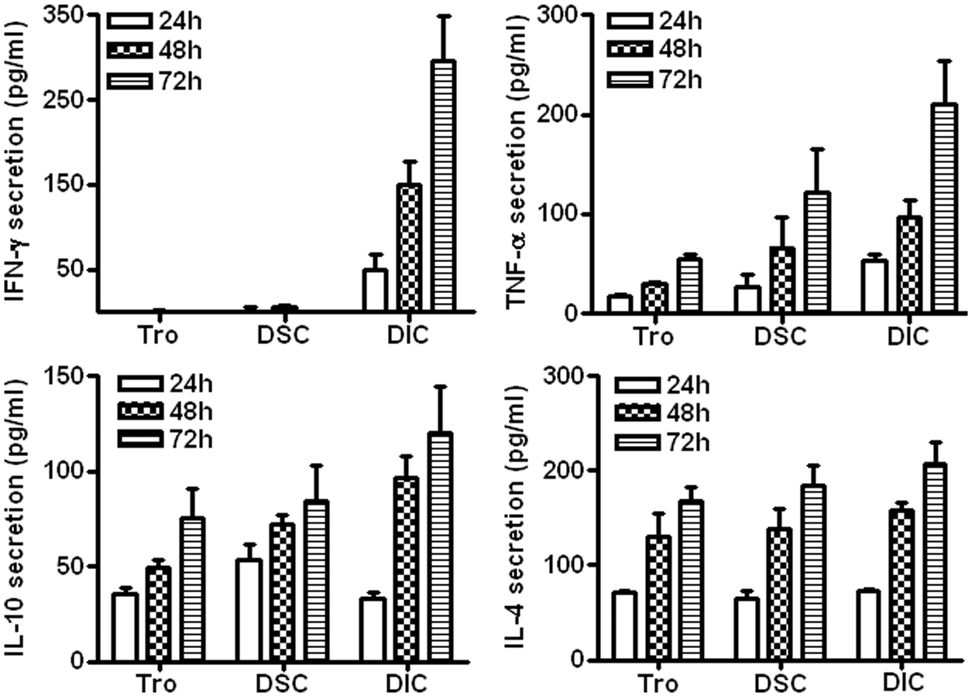
Secretion of cytokines in component cells at the maternal-fetal interface in early human pregnancy. Primary human first trimester trophoblasts, decidual stromal cells, or decidual immune cells were cultured in 24-well plates for 24, 48, or 72 h. The supernatants were harvested and subjected to a cytokine ELISA assay. Data represent the mean ± SE of three independent experiments with a total of 17 villi and three deciduas, performed in triplicate wells with three different samples.

Cells present at the maternal-fetal interface mainly include placental trophoblasts, DSCs and DICs. We first determined the Th1- and Th2-type cytokines secretion of these component cells at the maternal-fetal interface. Freshly isolated trophoblasts, DSCs or DICs were cultured in 24-well plates for 24–72 h, and the release of soluble cytokines into the culture supernatant was detected by using ELISA at 24-h intervals. As shown in figure 1, primary trophoblast cells secreted IL-4, IL-10, and TNF-α constitutively. At the end of 72 h, the accumulated concentrations of IL-4, IL-10, and TNF-α were 167.67±15.34, 75.27±15.73, and 54.26±4.65 pg/mL, respectively. Almost no IFN-γ secretion by trophoblasts was observed, even after 72 h of culture. Similarly, no IFN-γ secretion was seen in primary DSCs, and the secretion of IL-4, IL-10, and TNF-α was 183.63±21.81, 84.55±18.66, 120.93±43.42, respectively by DSCs after 72h of culture. On the other hand, DICs were the only cell types that produced IFN-γ at the maternal-fetal interface (about 294.73±54.23 pg/ml after 72 h of culture). DICs also produced about 209.51±43.58 pg/ml of TNF-α after 72 h of culture, which is much higher than that secreted by trophoblasts (P<0.05). DICs secreted similar levels of IL-4 (207.68±23.04 pg/ml) and IL-10 (120.23±24.26 pg/ml) to that of trophoblasts and DSCs (P>0.05, P>0.05, P>0.05, P>0.05).

### Characterization of Trophoblast Cells, DSCs, and DICs in Co-culture

**Figure 2:**
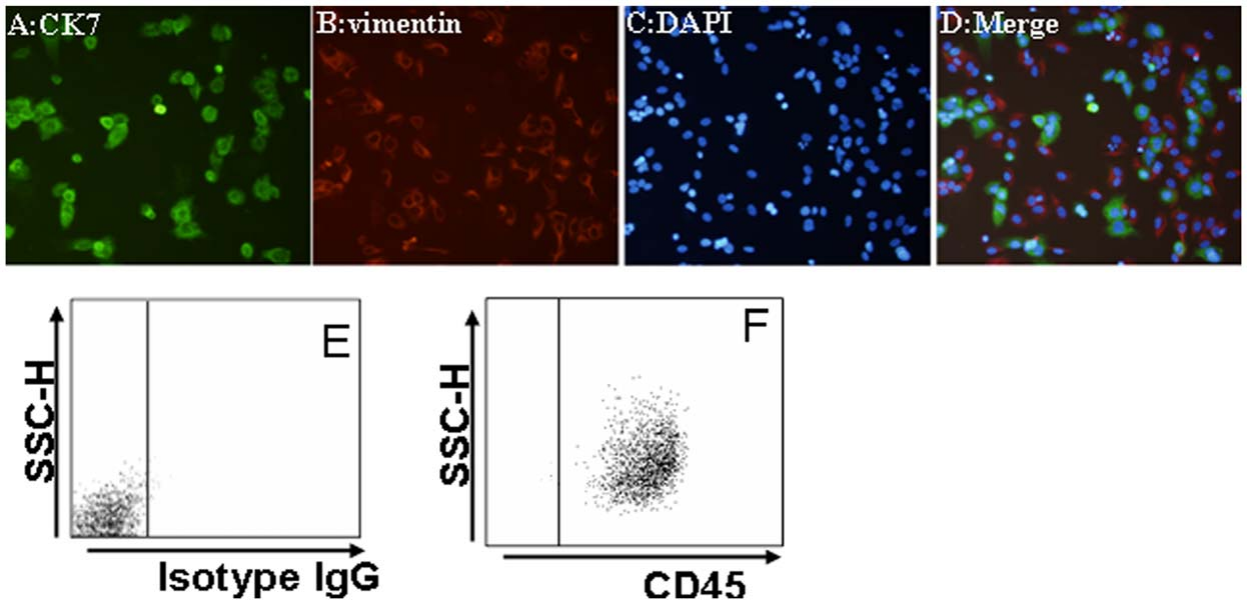
Characterization of cells in co-culture consisting of human primary trophoblasts, DSCs and DICs of early human pregnancy. In the co-culture, the attached trophoblasts (A) and DSCs (B) were stained and recognized by CK7 and vimentin antibodies, resulting in specific brown staining in the membrane and cytoplasm, respectively. No specific staining was seen with the isotype-control (C). Immune cells in suspension were harvested and stained with CD45-FITC (D) or the isotype control (E), and then subjected to flow cytometry. The percentages of positive cells in the indicated cells were shown. Data represent the mean ± SE of three independent experiments with 14 villi and three deciduas, performed in triplicate wells with three different samples.

To better simulate the maternal-fetal interface, we set up a co-culture with these three types of cells. In the co-culture, trophoblasts and DSCs were seeded on Matrigel-coated culture dishes, and DICs were then added. After 24 h of culture, we characterized trophoblasts by the marker CK7, and DSCs by the marker vimentin, among the attached cells by using immunofluorescence. Lymphocytes in suspension were harvested, and characterized by the CD45 marker with FCM. CD45 was originally called leukocyte common antigen, which is found on all leukocytes. Here, we chose CD45 as the common marker for characterization of DICs. As shown in Figure 2A, trophoblasts were stained with CK 7 but not vimentin, and were aggregated but not fused in Matrigel-coated wells. On the other hand, DSCs were stained by vimentin but not CK 7 around trophoblasts, as seen in Figure 2B. DAPI staining was observed in all the detected cells (Figure 2C). The merged picture showed us that trophoblasts and DSCs grew up well in the co-culture. (Figure 2D). CD45-positive cells accounted for more than 95% of total cells (Figure 2E, 2F). These results suggest that the co-culture to be used to mimic human maternal-fetal interface was successfully established.

### CsA Enhances Th2 Bias at the Human Maternal-fetal Interface

**Figure 3:**
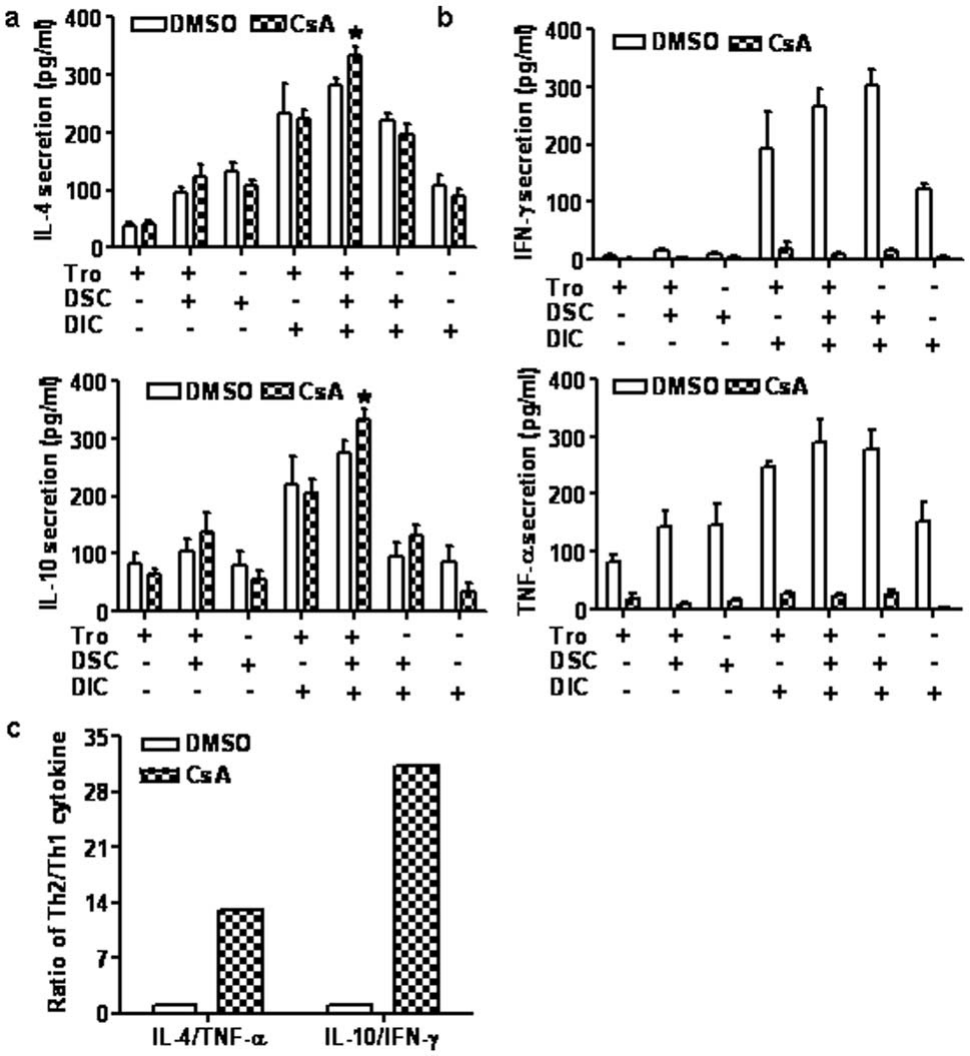
CsA promotes Th2 bias at the maternal-fetal interface in early human pregnancy. A: Primary human trophoblasts, DSCs, DICs, or various co-culture combinations were treated with CsA (1.0 µM) or DMSO (1/1000, v/v) for 48 h. Before harvest, the cells were treated with phorbol myristate acetate (PMA) and ionomycin for 4 h. The supernatants were harvested and subjected to ELISA. Cytokine secretion into the culture media is shown. Data represent the mean ± SE of three independent experiments with 23 villi and three deciduas, performed in triplicate wells with three different samples. B. The ratios of IL-4/TNF-α and IL-10/IFN-γ in the co-culture were obtained from the mean values in 3A, and presented in 3B.

Our previous study has demonstrated that administration with CsA in an abortion-prone matings led to a Th2 bias *in vivo* at the maternal-fetal interface [20]. To further investigate whether CsA regulates cytokine production *in vitro* at the maternal-fetal interface in early human pregnancy, we measured the secretion of these cytokines into the culture supernatant in individual cultures consisting of a single cell type, as well as in different co-culture combinations of trophoblasts, DSCs, and DICs. As shown in Figure 3a, treatment with CsA almost completely blocked IFN-γ and TNF-α secretion in all cell cultures. Unexpectedly, CsA increased the secretion of IL-4 and IL-10 in the co-culture of all three cell types. It was interesting that these increased levels of IL-4 and IL-10 were not observed in any of the respective cultures or in co-cultures of any two cell types, indicating that the increase in Th2 cytokine production by CsA occurs with the aid of the interaction among trophoblasts, DSCs, and DICs (Figure 3b). We further found that CsA increased the ratios of IL-4/TNF-α and IL-10/IFN-γ in the co-culture of trophoblasts, DSCs, and DICs (Figure 3c). Together, these results suggest that CsA enhances Th2 bias by harmonizing the crosstalk between maternal decidual cells and embryonic trophoblasts in early human pregnancy.

### CsA Promotes Th2-type but Inhibits Th1-type Cytokine Production in Component Cells at Human Maternal-fetal Interface

**Figure 4:**
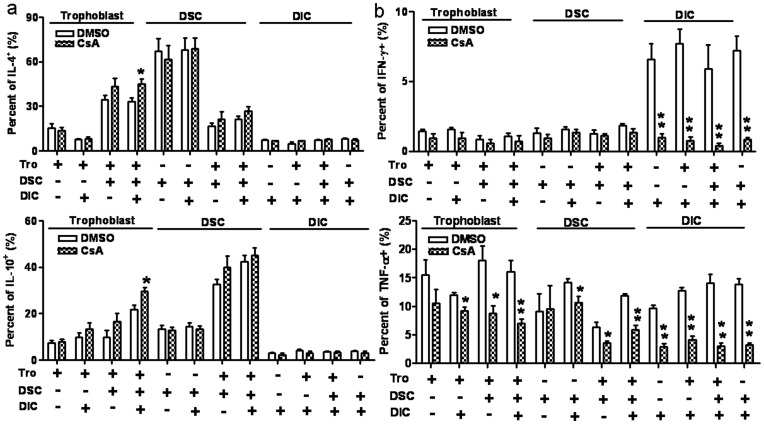
Effect of CsA on intracellular cytokine production in individual component cells at the maternal-fetal interface. Primary human trophoblasts, decidual stromal cells, decidual immune cells, or various co-culture combinations were treated with CsA (1.0 µM) or DMSO (1/1000, v/v) for 48 h. Before harvest, the cells were treated with PMA, and ionomycin, or with brefeldin A for 4 h. The cells were labeled for the surface expression of CD45 (FITC) and for intracellular cytokeratin 7, vimentin, TNF-α, IFN-γ, IL-4, and IL-10 using PE-conjugated mAbs. The intracellular production of TNF-α, IFN-γ, IL-4, and IL-10 was analyzed by flow cytometry. The percentages of positive cells among the indicated cells are shown. Data represent the mean ± SE of three independent experiments with 29 villi and three deciduas, performed in triplicate wells with three different samples. *P<0.05, compared to the vehicle control; Tro: Human primary trophoblasts.

To further define the contributions of the functional cells to the CsA-induced Th2 bias at the maternal-fetal interface, we investigated the intracellular production of cytokines by FCM. As shown in Figure 4A, the CsA-induced increase in intracellular IL-4 and IL-10 levels was seen in trophoblasts co-cultured with DSCs and DICs. Interestingly, CsA treatment did not affect the production of IL-4 or IL-10 in trophoblasts alone, or in trophoblasts co-cultured with either DSCs or DICs. Additionally, treatment with CsA did not affect the production of IL-4 and IL-10 in DSCs and DICs under any culture condition. These results suggested that CsA could increase Th2 cytokine production only in trophoblasts; however, the other cell types (DSCs and DICs) are also indispensable in the CsA-induced Th2 cytokine production at maternal-fetal interface.

In contrast, the inhibition of IFN-γ secretion by CsA at the maternal-fetal interface was due to decreased IFN-γ production in DICs, but not in trophoblasts or DSCs (Figure 4B). Although CsA could markedly decrease TNF-α production in DICs with or without other two types of cells (P<0.01, P<0.01 P<0.01 P<0.01), CsA did inhibit TNF-α production in trophoblasts and DSCs when co-cultured with another one or two types of cells (P<0.05, P<0.05, P<0.05, P<0.05), especially in mixed co-cultures of trophoblasts with DSCs and DICs (P<0.01). These data demonstrate that CsA promotes Th2 bias by up-regulating Th2-type cytokine production and down-regulating Th1-type cytokine production in different functional cells, and thereby modulating their crosstalk at the maternal-fetal interface in early human pregnancy.

## Discussion

CsA is a powerful immunosuppressant that is widely used to prevent organ rejection and to treat several autoimmune diseases [9], [25]. The clinical application of CsA has revolutionized organ transplantation, and improved the therapeutic management of some autoimmune diseases. From an immunological standpoint, the feto-placental unit is considered an allograft that resides in an immune competent mother. The decidual tissue that provides an attachment site for the placental trophoblast is heavily infiltrated with maternal lymphocytes in pregnancy. Interestingly, depletion of immune cells, rather than helping pregnancy, leads to termination of pregnancy [26]. Paradigms from transplantation immunology provide models for investigating maternal-fetal relationships, and for the development of prophylactics and therapeutics for pregnancy loss. Our previous data have also shown that administration of CsA induced Th2 bias at the maternal-fetal interface, and thereby led to improved pregnancy outcomes in abortion-prone mouse models [19]. Therefore, it is extremely meaningful to explore how CsA could induce Th2 bias at the maternal-fetal interface in early human pregnancy.

The cell constitution at the maternal-fetal interface is very complicated in early human pregnancy. Based on their origin, the main component cells at the maternal-fetal interface can be classified as embryonic (trophoblasts) or maternal (DSCs and DICs). These cells interact with each other to establish a unique immune milieu at the maternal-fetal interface by producing various regulatory factors that contribute to fetal survival and development in the maternal uterus until parturition. As a key component in the human placenta, embryo-derived trophoblasts play an important role in the shaping of the Th2 bias at the maternal-fetal interface [21], [22]. Pregnancy wastage is accompanied by the down-regulation of both pro- and anti-inflammatory cytokines in trophoblasts [27]. The regulatory cytokine IL-10 and the Th2-dominant cytokine IL-4 promote trophoblast invasion, and conduce to Th2 polarization and maternal-fetal immunotolerance. Trophoblasts produce cytokines such as IL-10 and TNF-α [28]. Cytokine gene polymorphisms in the promoter regions of TNF-α and IL-10 in trophoblasts are associated with recurrent pregnancy loss [29]. Placental trophoblasts inhibit the production of Th1-type cytokines and Th17-type cytokines by peripheral T lymphocytes. Trophoblasts also inhibit the expression of transcription factors that regulate Th1 immunity, and enhance the expression of transcription factors that regulate Th2 immunity [30]. Our previous studies have demonstrated that CsA promotes the biological functions of first-trimester cytotrophoblast cells in human pregnancy [31], [32], [33], [34], indicating that CsA might regulate the Th1/Th2 cytokine balance in trophoblasts.

DSCs are the major cellular component in decidua. In addition to secreting a series of cytokines, DSCs act as non-professional APCs and play a crucial role in the regulation of decidual CD4^+^ T-cell cytokine production, thereby helping maintain a balanced cytokine milieu at the maternal-fetal interface. DSCs can operate together with trophoblasts in modulating trophoblast invasion and placentation [1]. DSCs and trophoblasts produce macrophage inhibitory cytokine 1, which modulates the dendritic cell (DC) phenotype, resulting in a tolerogenic subtype of DCs in the decidua [23]. Our previous study has shown that CsA promotes the cross-talk between human cytotrophoblasts and DSCs by upregulating the CXCL12/CXCR4 interaction [34]. These data suggest that DSCs either alone or interacting with trophoblasts might be important regulators of the Th2 bias at the maternal-fetal interface.

Maternal lymphocytes are concentrated in the intervillous space at the maternal-fetal interface. It is well established that decidual lymphocytes play a major part in local cytokine production and Th2 bias in successful pregnancy. In contrast to peripheral blood, there is a unique composition of immune cells at the maternal-fetal interface, with approximately 50–70% natural killer cells, 15% macrophages, and 10% CD3^+^ T lymphocytes. These cells, when activated, produce a variety of cytokines and growth factors that participate in maternal-fetal tolerance, spiral artery/tissue remodeling, and placentation [2], [6], [35], [36].

In the present study, we have found that trophoblasts and DSCs express TNF-α, IL-4, and IL-10, but not IFN-γ. Treatment with CsA did not change the production of any of the examined cytokines in trophoblasts or DSCs alone. However, treatment with CsA decreases TNF-α production and increases IL-4 and IL-10 production in trophoblasts co-cultured with DSCs and DICs. CsA inhibits TNF-α production in DSCs when co-cultured with trophoblasts, but has no effect on IL-4 or IL-10 production in DSCs co-cultured with trophoblasts and/or DICs. In contrast, DICs produce all the examined cytokines; treatment with CsA completely inhibits Th1-type cytokine production but has no effect on Th2-type cytokine production in DICs either cultured alone or co-cultured with other cell types. These data suggest that the component cells at the maternal-fetal interface present various cytokine expression profiles. CsA can differently regulate cytokine production in these cells and induce a Th2 bias at the maternal-fetal interface. Furthermore, this induced Th2 bias by CsA at the maternal-fetal interface requires a coordinated interaction between embryonic trophoblasts and maternal DSCs and DICs. DSCs and DICs might amplify the effect of CsA on the Th2 bias in trophoblasts at the maternal-fetal interface. It is very interesting that different levels of cytokine production in a certain cell were observed when all cell types are co-cultured, suggesting that the cytokine production was regulated in the co-culture. Some soluble factors or surface molecules expressed on the other cells in the co-cultured system might play an important role in regulating cytokine production in the detecting cells. Our unpublished data showed that CXCR12/CXCR4 axis was one of the intermediaries involved in the crosstalk and cytokine production in the component cells at the maternal-fetal interface.

It is worth to note that there has been much concern over the potential detrimental effects of the immunosuppressive drug. To our knowledge, no report has been available about CsA usage for preventing pregnancy loss. There did have been a growing number of case reports on transplant patients receiving CsA who delivered a normal child. Indeed, most follow-up studies showed that the offspring appeared to have normal postnatal growth and development [37], [38]. Our previous studies demonstrated that CsA at low dosage could improve the biological function of human trophoblasts in early pregnancy [31], [32], [33], [34]. We also observed the improved pregnant outcomes of mouse abortion-prone models by low dosage of CsA only once during peri-implantation via promoting functions of trophoblasts and inducing maternal tolerance to the allogeneic fetus [19], [20]. This was consistent with the increasing evidence that the parameters featuring a maternal immune response during murine pregnancy are established early in gestation. It was reported that no significant passage of CsA into the fetus occurred after a single injection in pregnant mice [39]. This supports the possibility that a single injection of CsA during the peri-implantation period may prevent abortions and have no side effects on embryo development. Furthermore, the dosage of CsA used in our study is far lower than that used in transplantation. In addition, in our clinical study, we have used CsA to women experiencing recurrent spontaneous abortion. The primary results showed that taking low dosage of CsA orally (CsA concentration in peripheral blood is 50–100 ng/ml) at peri-implantation for around 20 days can efficiently prevent miscarriage (Data not shown). As the clinic follow-up is going on, we have not find any side effect on the mum and baby up to now. Therefore, the results are encouraging. However, more research is warranted to confirm these results by follow-up studies towards adulthood in a larger population.
